# (Naphthalene-2,3-diolato-κ^2^ *O*,*O*′)[tris­(pyridin-2-ylmeth­yl)amine-κ^4^ *N*]cobalt(III) tetra­phenyl­borate acetone monosolvate hemihydrate

**DOI:** 10.1107/S1600536812035210

**Published:** 2012-09-08

**Authors:** Fan Yu

**Affiliations:** a Key Laboratory of Optoelectronic Chemical Materials and Devices of the Ministry of Education, Jianghan University, Wuhan 430056, People’s Republic of China; b School of Chemical and Environmental Engineering, Jianghan University, Wuhan 430056, People’s Republic of China

## Abstract

In the title salt, [Co(C_10_H_6_O_2_)(C_18_H_18_N_4_)](C_24_H_20_B)·C_3_H_6_O·0.5H_2_O, the Co^III^ ion in the complex cation is six-coordinated in a rigid octa­hedral N_4_O_2_ geometry. The asymmetric unit contains one complete [Co(C_10_H_6_O_2_)(C_18_H_18_N_4_)]^+^ unit, one tetraphenylborate counter-anion and one acetone and one water mol­ecule that is located on an inversion centre. All the features of the Co^III^ ion are fully consistent with the formulation of the cation as a Co^3+^–catecholate complex. Variable-temperature magnetic measurements in the region 2–380 K show a obvious diamagnetism over the observed temperature range.

## Related literature

For related structures, see: Tinoco *et al.* (2008 ▶); Li *et al.* (2011 ▶); Guo *et al.* (2011 ▶); Tao *et al.* (2006 ▶). 
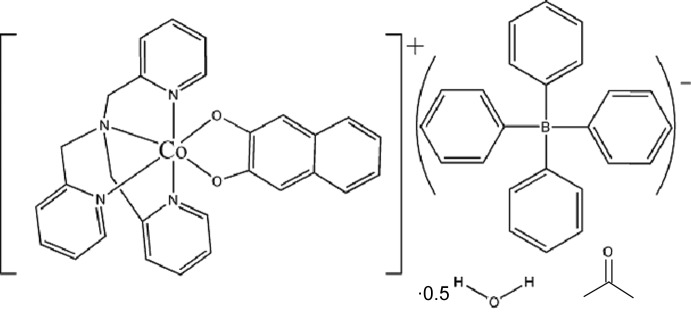


## Experimental

### 

#### Crystal data

[Co(C_10_H_6_O_2_)(C_18_H_18_N_4_)](C_24_H_20_B)·C_3_H_6_O·0.5H_2_O
*M* *_r_* = 893.74
Monoclinic, 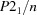

*a* = 17.2841 (2) Å
*b* = 12.2151 (2) Å
*c* = 21.7618 (3) Å
β = 94.449 (1)°
*V* = 4580.66 (11) Å^3^
*Z* = 4
Mo *K*α radiation
μ = 0.43 mm^−1^
*T* = 293 K
0.3 × 0.2 × 0.2 mm

#### Data collection

Oxford Gemini S Ultra diffractometer
Absorption correction: multi-scan (*CrysAlis RED*; Oxford Diffraction, 2006 ▶) *T*_min_ = 0.903, *T*_max_ = 0.918
38795 measured reflections
8732 independent reflections
6220 reflections with *I* > 2σ(*I*)
*R*_int_ = 0.036

#### Refinement

*R*[*F* ^2^ > 2σ(*F* ^2^)] = 0.044
*wR*(*F* ^2^) = 0.109
*S* = 1.07
8732 reflections
577 parameters
H-atom parameters constrained
Δρ_max_ = 1.28 e Å^−3^
Δρ_min_ = −0.68 e Å^−3^

### 

Data collection: *CrysAlis CCD* (Oxford Diffraction, 2006 ▶); cell refinement: *CrysAlis RED* (Oxford Diffraction, 2006 ▶); data reduction: *CrysAlis RED*; program(s) used to solve structure: *SHELXS97* (Sheldrick, 2008 ▶); program(s) used to refine structure: *SHELXL97* (Sheldrick, 2008 ▶); molecular graphics: *SHELXTL* (Sheldrick, 2008 ▶); software used to prepare material for publication: *publCIF* (Westrip, 2010) ▶.

## Supplementary Material

Crystal structure: contains datablock(s) I, global. DOI: 10.1107/S1600536812035210/hp2037sup1.cif

Structure factors: contains datablock(s) I. DOI: 10.1107/S1600536812035210/hp2037Isup2.hkl

Additional supplementary materials:  crystallographic information; 3D view; checkCIF report

## Figures and Tables

**Fig. 1.**
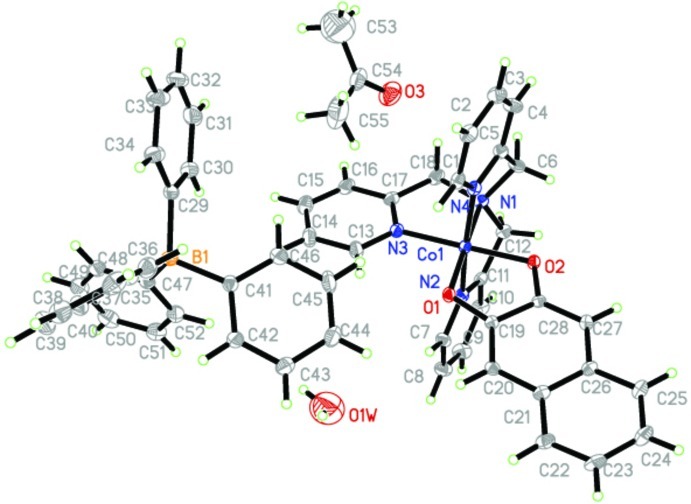
the asymmetric unit of the title compound 1 showing the atomic numbering and 30% probability displacement ellipsoids.

## supplementary crystallographic information

### Comment

Coordination complexes that catecholates (Cat) coordinate directly to cobalt or iron ions in high oxidation states, might exhibit interesting properties (Li *et al.*, 2010; Tao *et al.*,2006). Up to now, great effort have been devoted to search for new types of thus catecholates to construct more functional materials. Naphthalene-2,3-diol, acting as one kind of catecholates, possesses the bi-dentate chelate mode and much stronger π-conjugate systems. Complexes formed by the connection of transitional metals and naphthalene-2,3-diol have been synthesized and crystallographically characterized (Tinoco *et al.*, 2008), but rare cobalt ones documented (Guo *et al.*, 2011).

In this study, a new mononuclear Co compound with the tripodal ligand tris(2-pyridylmethyl)amine (tpa), naphthalene-2,3-diolate (ND) and counteranions, [Co(tpa)(ND)]BPh_4_ (1) has been prepared and structurally characterized.

### Experimental

To a well stirred methanol solution (20 ml) containing tpa (2.02 mmol) and CoCl_2_.6H_2_O(2.0 mmol) was added a methanol solution (10 ml) containing ND (0.5 mmol) and triethylamine (140 µ*L*) under inert atmosphere in methanol (15 ml). The resulting mixture was gently stirred at room temperature for 2 h and then NaBPh_4_ (0.5 mmol) was added for 1. The precipitation was dissolved in the mixture of acetone and water (5 ml/ 1 mL), and green crystals of compound 1 were obtained by slow evaporation of the filtrate.

### Refinement

All H atoms were placed geometrically with C—H = 0.93 (aromatic) or 0.96 Å (CH_2_), and refined using a riding atom model with their isotropic displacement factors, *U*_iso_ fixed at 1.2 time the *U*_eq_ of the parent C. The oxygen atom and hydrogen atoms on water molecule had been modeled over two sites with the sum of their respective occupancies equal to one.

### Crystal data

**Table:**

[Co(C_10_H_6_O_2_)(C_18_H_18_N_4_)](C_24_H_20_B)·C_3_H_6_O·0.5H_2_O	*F*(000) = 1876
*M**_r_* = 893.74	*D*_x_ = 1.296 Mg m^−^^3^*D*_m_ = 1.296 Mg m^−^^3^*D*_m_ measured by not measured
Monoclinic, *P*2_1_/*n*	Mo *K*α radiation, λ = 0.71073 Å
Hall symbol: -P 2yn	Cell parameters from 8994 reflections
*a* = 17.2841 (2) Å	θ = 0.9–0.9°
*b* = 12.2151 (2) Å	µ = 0.43 mm^−^^1^
*c* = 21.7618 (3) Å	*T* = 293 K
β = 94.449 (1)°	Block, green
*V* = 4580.66 (11) Å^3^	0.3 × 0.2 × 0.2 mm
*Z* = 4	

### Data collection

**Table:**

Oxford Gemini S Ultra diffractometer	8732 independent reflections
Radiation source: fine-focus sealed tube	6220 reflections with *I* > 2σ(*I*)
Graphite monochromator	*R*_int_ = 0.036
Detector resolution: 0 pixels mm^-1^	θ_max_ = 26.0°, θ_min_ = 2.2°
ω scans	*h* = −21→21
Absorption correction: multi-scan (*CrysAlis RED*; Oxford Diffraction, 2006)	*k* = −15→14
*T*_min_ = 0.903, *T*_max_ = 0.918	*l* = −26→26
38795 measured reflections	

### Refinement

**Table:**

Refinement on *F*^2^	Primary atom site location: structure-invariant direct methods
Least-squares matrix: full	Secondary atom site location: difference Fourier map
*R*[*F*^2^ > 2σ(*F*^2^)] = 0.044	Hydrogen site location: inferred from neighbouring sites
*wR*(*F*^2^) = 0.109	H-atom parameters constrained
*S* = 1.07	*w* = 1/[σ^2^(*F*_o_^2^) + (0.0589*P*)^2^] where *P* = (*F*_o_^2^ + 2*F*_c_^2^)/3
8732 reflections	(Δ/σ)_max_ = 0.005
577 parameters	Δρ_max_ = 1.28 e Å^−^^3^
0 restraints	Δρ_min_ = −0.68 e Å^−^^3^

### Special details

**Table:**

Experimental. Absorption correction: CrysAlis RED, Oxford Diffraction Ltd., Version 1.171.32.4 (release 27-04-2006 CrysAlis171 .NET) (compiled Apr 27 2007,17:53:11) Empirical absorption correction using spherical harmonics, implemented in SCALE3 ABSPACK scaling algorithm.
Geometry. All e.s.d.'s (except the e.s.d. in the dihedral angle between two l.s. planes) are estimated using the full covariance matrix. The cell e.s.d.'s are taken into account individually in the estimation of e.s.d.'s in distances, angles and torsion angles; correlations between e.s.d.'s in cell parameters are only used when they are defined by crystal symmetry. An approximate (isotropic) treatment of cell e.s.d.'s is used for estimating e.s.d.'s involving l.s. planes.
Refinement. Refinement of *F*^2^ against ALL reflections. The weighted *R*-factor *wR* and goodness of fit *S* are based on *F*^2^, conventional *R*-factors *R* are based on *F*, with *F* set to zero for negative *F*^2^. The threshold expression of *F*^2^ > σ(*F*^2^) is used only for calculating *R*-factors(gt) *etc*. and is not relevant to the choice of reflections for refinement. *R*-factors based on *F*^2^ are statistically about twice as large as those based on *F*, and *R*- factors based on ALL data will be even larger.

### Fractional atomic coordinates and isotropic or equivalent isotropic displacement parameters (Å^2^)

**Table:**

	*x*	*y*	*z*	*U*_iso_*/*U*_eq_	Occ. (<1)
Co1	0.648826 (16)	0.59663 (2)	0.735881 (13)	0.01947 (9)	
O1	0.66689 (8)	0.48998 (12)	0.67667 (7)	0.0236 (3)	
N1	0.67108 (10)	0.50507 (14)	0.80782 (8)	0.0227 (4)	
O2	0.75525 (8)	0.63165 (11)	0.73936 (7)	0.0218 (3)	
N3	0.53932 (10)	0.56765 (15)	0.73363 (9)	0.0245 (4)	
C7	0.61603 (13)	0.6924 (2)	0.61364 (11)	0.0287 (5)	
H7A	0.6166	0.6216	0.5979	0.034*	
C19	0.73890 (12)	0.50345 (16)	0.65856 (10)	0.0200 (5)	
C1	0.67312 (13)	0.39651 (17)	0.81117 (11)	0.0261 (5)	
H1A	0.6618	0.3558	0.7755	0.031*	
N4	0.63097 (11)	0.71061 (14)	0.79551 (8)	0.0226 (4)	
N2	0.62918 (10)	0.70789 (15)	0.67432 (8)	0.0239 (4)	
C16	0.42041 (14)	0.6255 (2)	0.77033 (12)	0.0370 (6)	
H16A	0.3936	0.6723	0.7948	0.044*	
C28	0.78855 (12)	0.57473 (16)	0.69630 (10)	0.0189 (5)	
C17	0.50007 (13)	0.63329 (19)	0.76994 (11)	0.0276 (5)	
C23	0.95617 (16)	0.4019 (2)	0.54312 (12)	0.0396 (6)	
H23A	0.9759	0.3630	0.5111	0.047*	
C12	0.65057 (14)	0.81610 (17)	0.76547 (11)	0.0269 (5)	
H12A	0.6229	0.8759	0.7832	0.032*	
H12B	0.7058	0.8303	0.7725	0.032*	
C22	0.87775 (15)	0.40262 (19)	0.54895 (11)	0.0317 (5)	
H22A	0.8449	0.3648	0.5205	0.038*	
C10	0.61316 (15)	0.9002 (2)	0.66006 (12)	0.0350 (6)	
H10A	0.6118	0.9704	0.6765	0.042*	
C27	0.86648 (13)	0.58154 (16)	0.68703 (10)	0.0227 (5)	
H27A	0.8991	0.6251	0.7127	0.027*	
C11	0.62848 (13)	0.80919 (18)	0.69805 (11)	0.0250 (5)	
C26	0.89676 (13)	0.52201 (17)	0.63821 (11)	0.0239 (5)	
C3	0.70738 (15)	0.40151 (19)	0.91895 (12)	0.0350 (6)	
H3A	0.7196	0.3665	0.9564	0.042*	
C20	0.76620 (13)	0.45262 (17)	0.60877 (10)	0.0228 (5)	
H20A	0.7323	0.4129	0.5820	0.027*	
C25	0.97789 (14)	0.51886 (19)	0.63125 (12)	0.0315 (6)	
H25A	1.0118	0.5577	0.6585	0.038*	
C8	0.60172 (15)	0.7789 (2)	0.57385 (12)	0.0367 (6)	
H8A	0.5933	0.7671	0.5316	0.044*	
C21	0.84594 (13)	0.45986 (17)	0.59752 (10)	0.0240 (5)	
C2	0.69153 (14)	0.34246 (19)	0.86593 (12)	0.0321 (6)	
H2A	0.6932	0.2664	0.8669	0.039*	
C13	0.50105 (14)	0.4926 (2)	0.69808 (12)	0.0333 (6)	
H13A	0.5287	0.4483	0.6727	0.040*	
C5	0.68636 (13)	0.56417 (18)	0.85977 (11)	0.0253 (5)	
C47	0.28443 (14)	0.1578 (2)	0.60310 (11)	0.0300 (5)	
C29	0.32966 (13)	0.0782 (2)	0.71392 (11)	0.0338 (6)	
C42	0.46483 (14)	0.10923 (19)	0.57230 (11)	0.0300 (5)	
H42A	0.4344	0.0726	0.5418	0.036*	
C41	0.43586 (13)	0.12134 (18)	0.63083 (11)	0.0267 (5)	
C52	0.30367 (15)	0.2526 (2)	0.57075 (12)	0.0334 (6)	
H52A	0.3557	0.2719	0.5709	0.040*	
B1	0.34765 (16)	0.0776 (2)	0.64037 (13)	0.0305 (6)	
C35	0.34366 (14)	−0.0491 (2)	0.61303 (11)	0.0299 (5)	
C48	0.20348 (14)	0.1387 (2)	0.60178 (12)	0.0363 (6)	
H48A	0.1862	0.0789	0.6233	0.044*	
C45	0.55778 (16)	0.2131 (2)	0.66083 (14)	0.0432 (7)	
H45A	0.5894	0.2478	0.6915	0.052*	
C18	0.54781 (14)	0.7077 (2)	0.81126 (12)	0.0329 (6)	
H18A	0.5458	0.6835	0.8536	0.040*	
H18B	0.5263	0.7810	0.8080	0.040*	
C4	0.70468 (16)	0.51565 (19)	0.91537 (11)	0.0339 (6)	
H4A	0.7153	0.5580	0.9505	0.041*	
C46	0.48541 (14)	0.1741 (2)	0.67457 (12)	0.0347 (6)	
H46A	0.4698	0.1837	0.7142	0.042*	
C6	0.68487 (15)	0.68640 (18)	0.85007 (11)	0.0282 (5)	
H6A	0.6675	0.7228	0.8862	0.034*	
H6B	0.7365	0.7127	0.8434	0.034*	
C43	0.53624 (14)	0.1490 (2)	0.55810 (12)	0.0351 (6)	
H43A	0.5523	0.1406	0.5186	0.042*	
C34	0.33037 (15)	−0.0146 (3)	0.75150 (12)	0.0410 (7)	
H34A	0.3403	−0.0824	0.7343	0.049*	
C40	0.28804 (16)	−0.0890 (2)	0.56920 (12)	0.0369 (6)	
H40A	0.2489	−0.0420	0.5537	0.044*	
C36	0.40001 (15)	−0.1261 (2)	0.63301 (13)	0.0377 (6)	
H36A	0.4395	−0.1040	0.6618	0.045*	
C51	0.24940 (16)	0.3187 (2)	0.53875 (12)	0.0392 (6)	
H51A	0.2656	0.3797	0.5176	0.047*	
C9	0.60013 (16)	0.8831 (2)	0.59778 (12)	0.0403 (7)	
H9A	0.5902	0.9424	0.5715	0.048*	
C44	0.58353 (15)	0.2015 (2)	0.60310 (13)	0.0410 (7)	
H44A	0.6318	0.2284	0.5944	0.049*	
C49	0.14908 (15)	0.2043 (2)	0.57029 (12)	0.0431 (7)	
H49A	0.0966	0.1876	0.5708	0.052*	
C24	1.00654 (15)	0.4589 (2)	0.58467 (13)	0.0387 (6)	
H24A	1.0597	0.4565	0.5809	0.046*	
C38	0.34353 (18)	−0.2691 (2)	0.56939 (14)	0.0456 (7)	
H38A	0.3429	−0.3412	0.5556	0.055*	
C37	0.40033 (17)	−0.2323 (2)	0.61265 (14)	0.0454 (7)	
H37A	0.4391	−0.2799	0.6281	0.054*	
C14	0.42237 (15)	0.4794 (2)	0.69812 (14)	0.0449 (7)	
H14A	0.3971	0.4252	0.6742	0.054*	
C32	0.30146 (16)	0.0871 (3)	0.84074 (13)	0.0590 (9)	
H32A	0.2932	0.0898	0.8824	0.071*	
C50	0.17167 (17)	0.2944 (2)	0.53808 (13)	0.0440 (7)	
H50A	0.1350	0.3380	0.5163	0.053*	
C15	0.38099 (15)	0.5479 (2)	0.73427 (14)	0.0456 (7)	
H15A	0.3274	0.5415	0.7342	0.055*	
C33	0.31679 (16)	−0.0098 (3)	0.81356 (14)	0.0533 (8)	
H33A	0.3182	−0.0738	0.8368	0.064*	
C39	0.28806 (19)	−0.1956 (2)	0.54744 (14)	0.0492 (7)	
H39A	0.2499	−0.2176	0.5175	0.059*	
C31	0.29827 (18)	0.1819 (3)	0.80555 (15)	0.0575 (9)	
H31A	0.2870	0.2487	0.8233	0.069*	
C30	0.31220 (16)	0.1764 (3)	0.74293 (12)	0.0443 (7)	
H30A	0.3097	0.2406	0.7198	0.053*	
O3	0.50570 (14)	0.50146 (19)	0.89485 (11)	0.0679 (7)	
C54	0.47702 (16)	0.4131 (3)	0.90009 (15)	0.0487 (8)	
C53	0.4346 (4)	0.3874 (5)	0.9557 (3)	0.1506 (16)	
H53A	0.4368	0.4495	0.9828	0.226*	
H53B	0.4584	0.3255	0.9768	0.226*	
H53C	0.3814	0.3708	0.9432	0.226*	
C55	0.4813 (3)	0.3297 (4)	0.8537 (2)	0.1009 (16)	
H55A	0.5101	0.3569	0.8210	0.151*	
H55D	0.4298	0.3104	0.8376	0.151*	
H55B	0.5068	0.2662	0.8717	0.151*	
O1W	0.5000	0.5000	0.5000	0.1506 (16)	
H1WA	0.5158	0.4350	0.4948	0.226*	0.50
H1WB	0.4550	0.4983	0.5131	0.226*	0.50

### Atomic displacement parameters (Å^2^)

**Table:**

	*U*^11^	*U*^22^	*U*^33^	*U*^12^	*U*^13^	*U*^23^
Co1	0.01869 (15)	0.01865 (16)	0.02129 (16)	−0.00085 (12)	0.00289 (11)	−0.00366 (13)
O1	0.0196 (8)	0.0247 (8)	0.0267 (8)	−0.0032 (6)	0.0036 (6)	−0.0073 (7)
N1	0.0212 (9)	0.0209 (10)	0.0265 (10)	−0.0003 (8)	0.0046 (8)	−0.0038 (8)
O2	0.0192 (8)	0.0218 (7)	0.0246 (8)	−0.0008 (6)	0.0030 (6)	−0.0042 (6)
N3	0.0223 (10)	0.0262 (10)	0.0254 (10)	−0.0012 (8)	0.0033 (8)	−0.0012 (8)
C7	0.0247 (12)	0.0365 (13)	0.0244 (12)	0.0074 (10)	−0.0013 (10)	−0.0033 (11)
C19	0.0208 (11)	0.0181 (11)	0.0211 (11)	0.0034 (9)	0.0013 (9)	0.0028 (9)
C1	0.0247 (12)	0.0204 (11)	0.0338 (13)	−0.0025 (10)	0.0057 (10)	−0.0055 (10)
N4	0.0268 (10)	0.0179 (9)	0.0240 (10)	−0.0003 (8)	0.0071 (8)	−0.0023 (8)
N2	0.0188 (9)	0.0263 (10)	0.0268 (11)	0.0035 (8)	0.0035 (8)	−0.0020 (8)
C16	0.0266 (13)	0.0471 (15)	0.0386 (15)	0.0047 (12)	0.0115 (11)	−0.0002 (12)
C28	0.0233 (11)	0.0153 (10)	0.0185 (11)	0.0016 (8)	0.0036 (9)	0.0031 (8)
C17	0.0270 (12)	0.0298 (12)	0.0268 (13)	0.0046 (10)	0.0071 (10)	0.0037 (10)
C23	0.0462 (16)	0.0377 (14)	0.0381 (15)	0.0024 (13)	0.0239 (13)	−0.0029 (12)
C12	0.0328 (13)	0.0182 (11)	0.0304 (13)	0.0000 (10)	0.0070 (10)	−0.0003 (10)
C22	0.0404 (14)	0.0264 (12)	0.0299 (13)	−0.0033 (11)	0.0119 (11)	0.0010 (11)
C10	0.0384 (14)	0.0274 (13)	0.0398 (15)	0.0108 (11)	0.0076 (12)	0.0034 (12)
C27	0.0232 (11)	0.0176 (11)	0.0274 (12)	0.0002 (9)	0.0013 (9)	0.0008 (9)
C11	0.0207 (11)	0.0249 (12)	0.0301 (13)	0.0020 (9)	0.0063 (10)	0.0011 (10)
C26	0.0263 (12)	0.0190 (11)	0.0269 (12)	0.0010 (9)	0.0061 (10)	0.0047 (9)
C3	0.0422 (15)	0.0291 (13)	0.0340 (14)	0.0004 (11)	0.0057 (11)	0.0059 (12)
C20	0.0275 (12)	0.0204 (11)	0.0201 (12)	−0.0004 (9)	−0.0004 (9)	0.0015 (9)
C25	0.0254 (12)	0.0287 (12)	0.0415 (15)	−0.0002 (10)	0.0086 (11)	0.0014 (11)
C8	0.0363 (14)	0.0457 (16)	0.0279 (14)	0.0098 (12)	0.0021 (11)	0.0016 (12)
C21	0.0308 (12)	0.0160 (11)	0.0257 (12)	0.0025 (9)	0.0061 (10)	0.0069 (9)
C2	0.0365 (14)	0.0203 (12)	0.0401 (15)	−0.0006 (10)	0.0062 (12)	0.0039 (11)
C13	0.0268 (13)	0.0359 (13)	0.0368 (15)	−0.0023 (11)	0.0002 (11)	−0.0088 (12)
C5	0.0291 (12)	0.0224 (12)	0.0247 (12)	−0.0013 (9)	0.0034 (10)	−0.0036 (9)
C47	0.0278 (13)	0.0384 (14)	0.0240 (12)	0.0005 (11)	0.0041 (10)	−0.0100 (11)
C29	0.0211 (12)	0.0571 (17)	0.0230 (12)	−0.0055 (11)	0.0013 (10)	−0.0005 (12)
C42	0.0282 (12)	0.0343 (13)	0.0272 (13)	−0.0001 (11)	0.0012 (10)	0.0019 (11)
C41	0.0252 (12)	0.0273 (12)	0.0272 (12)	0.0007 (10)	−0.0007 (10)	0.0020 (10)
C52	0.0327 (13)	0.0328 (13)	0.0353 (14)	0.0030 (11)	0.0061 (11)	−0.0051 (11)
B1	0.0276 (14)	0.0423 (17)	0.0217 (13)	−0.0022 (12)	0.0031 (11)	−0.0009 (12)
C35	0.0276 (13)	0.0392 (14)	0.0234 (12)	−0.0047 (11)	0.0060 (10)	0.0044 (11)
C48	0.0297 (14)	0.0489 (15)	0.0310 (14)	−0.0002 (12)	0.0064 (11)	−0.0071 (12)
C45	0.0347 (15)	0.0455 (16)	0.0471 (17)	−0.0138 (12)	−0.0116 (13)	0.0021 (13)
C18	0.0323 (13)	0.0306 (13)	0.0380 (14)	−0.0016 (11)	0.0158 (11)	−0.0084 (11)
C4	0.0490 (16)	0.0283 (13)	0.0247 (13)	0.0007 (11)	0.0042 (11)	−0.0025 (10)
C46	0.0343 (14)	0.0412 (14)	0.0283 (14)	0.0000 (12)	−0.0007 (11)	0.0010 (11)
C6	0.0414 (14)	0.0215 (12)	0.0213 (12)	−0.0014 (10)	−0.0002 (10)	−0.0054 (10)
C43	0.0316 (14)	0.0399 (14)	0.0343 (14)	0.0031 (12)	0.0064 (11)	0.0075 (12)
C34	0.0280 (13)	0.0637 (18)	0.0309 (15)	0.0062 (13)	0.0003 (11)	0.0094 (13)
C40	0.0428 (15)	0.0328 (14)	0.0338 (14)	−0.0040 (12)	−0.0053 (12)	0.0034 (12)
C36	0.0284 (13)	0.0431 (15)	0.0418 (15)	0.0000 (11)	0.0045 (12)	0.0028 (12)
C51	0.0431 (16)	0.0355 (14)	0.0398 (16)	0.0106 (12)	0.0077 (12)	−0.0046 (12)
C9	0.0436 (16)	0.0415 (16)	0.0357 (15)	0.0159 (12)	0.0017 (12)	0.0140 (12)
C44	0.0274 (13)	0.0473 (16)	0.0481 (17)	−0.0059 (12)	0.0010 (12)	0.0155 (13)
C49	0.0262 (13)	0.0652 (19)	0.0379 (16)	0.0087 (13)	0.0022 (12)	−0.0105 (14)
C24	0.0287 (13)	0.0391 (15)	0.0504 (17)	0.0024 (12)	0.0163 (12)	−0.0008 (13)
C38	0.0572 (19)	0.0302 (14)	0.0512 (18)	−0.0027 (13)	0.0151 (15)	−0.0014 (13)
C37	0.0393 (16)	0.0427 (16)	0.0559 (19)	0.0086 (13)	0.0144 (14)	0.0129 (14)
C14	0.0284 (14)	0.0556 (17)	0.0503 (18)	−0.0110 (13)	0.0005 (12)	−0.0117 (14)
C32	0.0321 (15)	0.120 (3)	0.0253 (15)	−0.0012 (18)	0.0038 (12)	0.0025 (19)
C50	0.0428 (16)	0.0501 (17)	0.0382 (16)	0.0230 (14)	−0.0019 (13)	−0.0107 (14)
C15	0.0222 (13)	0.0664 (19)	0.0485 (18)	0.0011 (13)	0.0048 (12)	0.0009 (15)
C33	0.0326 (15)	0.094 (2)	0.0333 (16)	0.0104 (16)	0.0031 (12)	0.0169 (17)
C39	0.0560 (19)	0.0449 (17)	0.0453 (17)	−0.0075 (14)	−0.0058 (14)	−0.0040 (14)
C31	0.0422 (17)	0.087 (2)	0.0445 (18)	−0.0122 (17)	0.0106 (14)	−0.0259 (18)
C30	0.0401 (16)	0.0610 (18)	0.0328 (15)	−0.0110 (14)	0.0094 (12)	−0.0093 (14)
O3	0.0649 (15)	0.0596 (14)	0.0796 (18)	−0.0097 (12)	0.0088 (13)	0.0205 (13)
C54	0.0298 (14)	0.0501 (18)	0.066 (2)	−0.0034 (14)	0.0017 (14)	0.0175 (16)
C53	0.174 (4)	0.149 (3)	0.131 (4)	−0.024 (3)	0.019 (3)	−0.017 (3)
C55	0.070 (3)	0.099 (3)	0.136 (4)	−0.033 (2)	0.027 (3)	−0.035 (3)
O1W	0.174 (4)	0.149 (3)	0.131 (4)	−0.024 (3)	0.019 (3)	−0.017 (3)

### Geometric parameters (Å, º)

**Table:**

Co1—O1	1.8755 (14)	C42—C43	1.384 (3)
Co1—O2	1.8843 (14)	C42—C41	1.412 (3)
Co1—N2	1.9200 (19)	C42—H42A	0.9300
Co1—N3	1.9224 (18)	C41—C46	1.389 (3)
Co1—N1	1.9381 (19)	C41—B1	1.644 (3)
Co1—N4	1.9441 (17)	C52—C51	1.384 (4)
O1—C19	1.345 (3)	C52—H52A	0.9300
N1—C1	1.328 (3)	B1—C35	1.657 (4)
N1—C5	1.350 (3)	C35—C40	1.389 (4)
O2—C28	1.333 (2)	C35—C36	1.399 (4)
N3—C13	1.341 (3)	C48—C49	1.376 (4)
N3—C17	1.346 (3)	C48—H48A	0.9300
C7—N2	1.336 (3)	C45—C44	1.372 (4)
C7—C8	1.376 (3)	C45—C46	1.393 (4)
C7—H7A	0.9300	C45—H45A	0.9300
C19—C20	1.364 (3)	C18—H18A	0.9700
C19—C28	1.435 (3)	C18—H18B	0.9700
C1—C2	1.378 (3)	C4—H4A	0.9300
C1—H1A	0.9300	C46—H46A	0.9300
N4—C6	1.481 (3)	C6—H6A	0.9700
N4—C12	1.496 (3)	C6—H6B	0.9700
N4—C18	1.504 (3)	C43—C44	1.383 (4)
N2—C11	1.341 (3)	C43—H43A	0.9300
C16—C15	1.377 (4)	C34—C33	1.390 (4)
C16—C17	1.381 (3)	C34—H34A	0.9300
C16—H16A	0.9300	C40—C39	1.385 (4)
C28—C27	1.380 (3)	C40—H40A	0.9300
C17—C18	1.483 (3)	C36—C37	1.371 (4)
C23—C22	1.371 (4)	C36—H36A	0.9300
C23—C24	1.392 (4)	C51—C50	1.375 (4)
C23—H23A	0.9300	C51—H51A	0.9300
C12—C11	1.490 (3)	C9—H9A	0.9300
C12—H12A	0.9700	C44—H44A	0.9300
C12—H12B	0.9700	C49—C50	1.377 (4)
C22—C21	1.414 (3)	C49—H49A	0.9300
C22—H22A	0.9300	C24—H24A	0.9300
C10—C9	1.373 (4)	C38—C39	1.372 (4)
C10—C11	1.398 (3)	C38—C37	1.382 (4)
C10—H10A	0.9300	C38—H38A	0.9300
C27—C26	1.420 (3)	C37—H37A	0.9300
C27—H27A	0.9300	C14—C15	1.385 (4)
C26—C25	1.422 (3)	C14—H14A	0.9300
C26—C21	1.418 (3)	C32—C33	1.358 (5)
C3—C2	1.370 (4)	C32—C31	1.387 (5)
C3—C4	1.397 (3)	C32—H32A	0.9300
C3—H3A	0.9300	C50—H50A	0.9300
C20—C21	1.421 (3)	C15—H15A	0.9300
C20—H20A	0.9300	C33—H33A	0.9300
C25—C24	1.373 (4)	C39—H39A	0.9300
C25—H25A	0.9300	C31—C30	1.404 (4)
C8—C9	1.376 (4)	C31—H31A	0.9300
C8—H8A	0.9300	C30—H30A	0.9300
C2—H2A	0.9300	O3—C54	1.197 (3)
C13—C14	1.369 (4)	C54—C55	1.439 (5)
C13—H13A	0.9300	C54—C53	1.496 (7)
C5—C4	1.362 (3)	C53—H53A	0.9600
C5—C6	1.508 (3)	C53—H53B	0.9600
C47—C52	1.409 (3)	C53—H53C	0.9600
C47—C48	1.416 (3)	C55—H55A	0.9600
C47—B1	1.635 (4)	C55—H55D	0.9600
C29—C34	1.397 (4)	C55—H55B	0.9600
C29—C30	1.400 (4)	O1W—H1WA	0.8500
C29—B1	1.654 (4)	O1W—H1WB	0.8501
			
O1—Co1—O2	88.36 (6)	C51—C52—C47	123.6 (2)
O1—Co1—N2	92.42 (7)	C51—C52—H52A	118.2
O2—Co1—N2	89.23 (7)	C47—C52—H52A	118.2
O1—Co1—N3	94.05 (7)	C47—B1—C35	111.9 (2)
O2—Co1—N3	177.35 (7)	C47—B1—C41	109.5 (2)
N2—Co1—N3	89.55 (8)	C35—B1—C41	105.75 (19)
O1—Co1—N1	96.94 (7)	C47—B1—C29	108.0 (2)
O2—Co1—N1	88.08 (7)	C35—B1—C29	110.2 (2)
N2—Co1—N1	170.18 (8)	C41—B1—C29	111.5 (2)
N3—Co1—N1	92.73 (8)	C40—C35—C36	113.9 (2)
O1—Co1—N4	178.26 (7)	C40—C35—B1	125.6 (2)
O2—Co1—N4	90.90 (7)	C36—C35—B1	120.5 (2)
N2—Co1—N4	86.00 (8)	C49—C48—C47	123.4 (3)
N3—Co1—N4	86.67 (8)	C49—C48—H48A	118.3
N1—Co1—N4	84.60 (7)	C47—C48—H48A	118.3
C19—O1—Co1	108.88 (12)	C44—C45—C46	121.4 (2)
C1—N1—C5	119.0 (2)	C44—C45—H45A	119.3
C1—N1—Co1	128.55 (16)	C46—C45—H45A	119.3
C5—N1—Co1	112.43 (14)	C17—C18—N4	111.90 (18)
C28—O2—Co1	109.07 (13)	C17—C18—H18A	109.2
C13—N3—C17	119.9 (2)	N4—C18—H18A	109.2
C13—N3—Co1	125.54 (16)	C17—C18—H18B	109.2
C17—N3—Co1	114.55 (15)	N4—C18—H18B	109.2
N2—C7—C8	121.4 (2)	H18A—C18—H18B	107.9
N2—C7—H7A	119.3	C5—C4—C3	119.2 (2)
C8—C7—H7A	119.3	C5—C4—H4A	120.4
O1—C19—C20	124.20 (19)	C3—C4—H4A	120.4
O1—C19—C28	115.64 (18)	C45—C46—C41	121.9 (2)
C20—C19—C28	120.1 (2)	C45—C46—H46A	119.1
N1—C1—C2	121.9 (2)	C41—C46—H46A	119.1
N1—C1—H1A	119.0	N4—C6—C5	108.22 (18)
C2—C1—H1A	119.0	N4—C6—H6A	110.1
C6—N4—C12	111.88 (17)	C5—C6—H6A	110.1
C6—N4—C18	111.44 (18)	N4—C6—H6B	110.1
C12—N4—C18	111.91 (17)	C5—C6—H6B	110.1
C6—N4—Co1	105.64 (13)	H6A—C6—H6B	108.4
C12—N4—Co1	105.79 (13)	C44—C43—C42	119.5 (2)
C18—N4—Co1	109.82 (13)	C44—C43—H43A	120.2
C7—N2—C11	120.4 (2)	C42—C43—H43A	120.2
C7—N2—Co1	126.63 (16)	C29—C34—C33	122.7 (3)
C11—N2—Co1	113.00 (15)	C29—C34—H34A	118.7
C15—C16—C17	119.6 (2)	C33—C34—H34A	118.7
C15—C16—H16A	120.2	C35—C40—C39	123.1 (3)
C17—C16—H16A	120.2	C35—C40—H40A	118.4
O2—C28—C27	123.44 (19)	C39—C40—H40A	118.4
O2—C28—C19	116.56 (18)	C37—C36—C35	123.8 (3)
C27—C28—C19	120.01 (19)	C37—C36—H36A	118.1
N3—C17—C16	120.7 (2)	C35—C36—H36A	118.1
N3—C17—C18	116.0 (2)	C50—C51—C52	120.2 (3)
C16—C17—C18	123.1 (2)	C50—C51—H51A	119.9
C22—C23—C24	120.6 (2)	C52—C51—H51A	119.9
C22—C23—H23A	119.7	C10—C9—C8	120.5 (2)
C24—C23—H23A	119.7	C10—C9—H9A	119.7
C11—C12—N4	109.41 (18)	C8—C9—H9A	119.7
C11—C12—H12A	109.8	C43—C44—C45	118.7 (2)
N4—C12—H12A	109.8	C43—C44—H44A	120.7
C11—C12—H12B	109.8	C45—C44—H44A	120.7
N4—C12—H12B	109.8	C50—C49—C48	120.6 (3)
H12A—C12—H12B	108.2	C50—C49—H49A	119.7
C23—C22—C21	121.1 (2)	C48—C49—H49A	119.7
C23—C22—H22A	119.4	C25—C24—C23	120.2 (2)
C21—C22—H22A	119.4	C25—C24—H24A	119.9
C9—C10—C11	118.1 (2)	C23—C24—H24A	119.9
C9—C10—H10A	120.9	C39—C38—C37	117.7 (3)
C11—C10—H10A	120.9	C39—C38—H38A	121.2
C28—C27—C26	119.9 (2)	C37—C38—H38A	121.2
C28—C27—H27A	120.0	C38—C37—C36	120.5 (3)
C26—C27—H27A	120.0	C38—C37—H37A	119.8
N2—C11—C10	120.9 (2)	C36—C37—H37A	119.8
N2—C11—C12	114.98 (19)	C13—C14—C15	119.1 (3)
C10—C11—C12	124.0 (2)	C13—C14—H14A	120.5
C25—C26—C21	119.1 (2)	C15—C14—H14A	120.5
C25—C26—C27	121.1 (2)	C33—C32—C31	119.2 (3)
C21—C26—C27	119.7 (2)	C33—C32—H32A	120.4
C2—C3—C4	118.3 (2)	C31—C32—H32A	120.4
C2—C3—H3A	120.8	C49—C50—C51	118.8 (3)
C4—C3—H3A	120.8	C49—C50—H50A	120.6
C19—C20—C21	120.6 (2)	C51—C50—H50A	120.6
C19—C20—H20A	119.7	C16—C15—C14	119.0 (2)
C21—C20—H20A	119.7	C16—C15—H15A	120.5
C24—C25—C26	120.5 (2)	C14—C15—H15A	120.5
C24—C25—H25A	119.7	C32—C33—C34	120.9 (3)
C26—C25—H25A	119.7	C32—C33—H33A	119.5
C7—C8—C9	118.7 (2)	C34—C33—H33A	119.5
C7—C8—H8A	120.7	C38—C39—C40	121.0 (3)
C9—C8—H8A	120.7	C38—C39—H39A	119.5
C22—C21—C20	122.5 (2)	C40—C39—H39A	119.5
C22—C21—C26	118.3 (2)	C32—C31—C30	119.6 (3)
C20—C21—C26	119.2 (2)	C32—C31—H31A	120.2
C3—C2—C1	119.6 (2)	C30—C31—H31A	120.2
C3—C2—H2A	120.2	C29—C30—C31	122.5 (3)
C1—C2—H2A	120.2	C29—C30—H30A	118.7
N3—C13—C14	121.7 (2)	C31—C30—H30A	118.7
N3—C13—H13A	119.2	O3—C54—C55	121.8 (3)
C14—C13—H13A	119.2	O3—C54—C53	119.9 (4)
N1—C5—C4	121.9 (2)	C55—C54—C53	118.3 (4)
N1—C5—C6	114.34 (19)	C54—C53—H53A	109.5
C4—C5—C6	123.7 (2)	C54—C53—H53B	109.5
C52—C47—C48	113.3 (2)	H53A—C53—H53B	109.5
C52—C47—B1	124.5 (2)	C54—C53—H53C	109.5
C48—C47—B1	122.2 (2)	H53A—C53—H53C	109.5
C34—C29—C30	115.1 (2)	H53B—C53—H53C	109.5
C34—C29—B1	124.7 (2)	C54—C55—H55A	109.5
C30—C29—B1	120.2 (2)	C54—C55—H55D	109.5
C43—C42—C41	123.3 (2)	H55A—C55—H55D	109.5
C43—C42—H42A	118.3	C54—C55—H55B	109.5
C41—C42—H42A	118.3	H55A—C55—H55B	109.5
C46—C41—C42	115.1 (2)	H55D—C55—H55B	109.5
C46—C41—B1	126.4 (2)	H1WA—O1W—H1WB	109.5
C42—C41—B1	118.5 (2)		
			
O2—Co1—O1—C19	−10.58 (13)	N2—C7—C8—C9	−0.8 (4)
N2—Co1—O1—C19	78.58 (14)	C23—C22—C21—C20	−173.1 (2)
N3—Co1—O1—C19	168.29 (13)	C23—C22—C21—C26	3.6 (3)
N1—Co1—O1—C19	−98.45 (13)	C19—C20—C21—C22	175.7 (2)
N4—Co1—O1—C19	54 (3)	C19—C20—C21—C26	−0.9 (3)
O1—Co1—N1—C1	−18.5 (2)	C25—C26—C21—C22	−4.0 (3)
O2—Co1—N1—C1	−106.63 (19)	C27—C26—C21—C22	179.25 (19)
N2—Co1—N1—C1	179 (24)	C25—C26—C21—C20	172.7 (2)
N3—Co1—N1—C1	75.90 (19)	C27—C26—C21—C20	−4.0 (3)
N4—Co1—N1—C1	162.3 (2)	C4—C3—C2—C1	−0.4 (4)
O1—Co1—N1—C5	161.17 (15)	N1—C1—C2—C3	0.8 (4)
O2—Co1—N1—C5	73.06 (15)	C17—N3—C13—C14	−0.9 (4)
N2—Co1—N1—C5	−1.1 (5)	Co1—N3—C13—C14	−178.1 (2)
N3—Co1—N1—C5	−104.41 (16)	C1—N1—C5—C4	0.8 (3)
N4—Co1—N1—C5	−18.02 (15)	Co1—N1—C5—C4	−178.93 (19)
O1—Co1—O2—C28	5.73 (13)	C1—N1—C5—C6	178.1 (2)
N2—Co1—O2—C28	−86.71 (13)	Co1—N1—C5—C6	−1.6 (2)
N3—Co1—O2—C28	−149.3 (16)	C43—C42—C41—C46	−1.7 (3)
N1—Co1—O2—C28	102.74 (13)	C43—C42—C41—B1	175.9 (2)
N4—Co1—O2—C28	−172.69 (13)	C48—C47—C52—C51	−2.4 (3)
O1—Co1—N3—C13	0.0 (2)	B1—C47—C52—C51	179.1 (2)
O2—Co1—N3—C13	154.9 (15)	C52—C47—B1—C35	−119.9 (2)
N2—Co1—N3—C13	92.4 (2)	C48—C47—B1—C35	61.6 (3)
N1—Co1—N3—C13	−97.2 (2)	C52—C47—B1—C41	−3.0 (3)
N4—Co1—N3—C13	178.4 (2)	C48—C47—B1—C41	178.5 (2)
O1—Co1—N3—C17	−177.43 (16)	C52—C47—B1—C29	118.5 (2)
O2—Co1—N3—C17	−22.5 (17)	C48—C47—B1—C29	−59.9 (3)
N2—Co1—N3—C17	−85.04 (16)	C46—C41—B1—C47	106.6 (3)
N1—Co1—N3—C17	85.41 (16)	C42—C41—B1—C47	−70.7 (3)
N4—Co1—N3—C17	0.98 (16)	C46—C41—B1—C35	−132.6 (2)
Co1—O1—C19—C20	−168.23 (17)	C42—C41—B1—C35	50.0 (3)
Co1—O1—C19—C28	13.2 (2)	C46—C41—B1—C29	−12.8 (3)
C5—N1—C1—C2	−1.0 (3)	C42—C41—B1—C29	169.8 (2)
Co1—N1—C1—C2	178.65 (17)	C34—C29—B1—C47	137.4 (2)
O1—Co1—N4—C6	−120 (2)	C30—C29—B1—C47	−42.7 (3)
O2—Co1—N4—C6	−55.71 (14)	C34—C29—B1—C35	14.8 (3)
N2—Co1—N4—C6	−144.87 (14)	C30—C29—B1—C35	−165.2 (2)
N3—Co1—N4—C6	125.35 (14)	C34—C29—B1—C41	−102.3 (3)
N1—Co1—N4—C6	32.28 (14)	C30—C29—B1—C41	77.7 (3)
O1—Co1—N4—C12	−2 (3)	C47—B1—C35—C40	−7.7 (3)
O2—Co1—N4—C12	63.06 (14)	C41—B1—C35—C40	−126.8 (2)
N2—Co1—N4—C12	−26.10 (14)	C29—B1—C35—C40	112.5 (3)
N3—Co1—N4—C12	−115.88 (14)	C47—B1—C35—C36	171.2 (2)
N1—Co1—N4—C12	151.05 (15)	C41—B1—C35—C36	52.1 (3)
O1—Co1—N4—C18	119 (2)	C29—B1—C35—C36	−68.6 (3)
O2—Co1—N4—C18	−175.99 (15)	C52—C47—C48—C49	2.1 (4)
N2—Co1—N4—C18	94.84 (15)	B1—C47—C48—C49	−179.3 (2)
N3—Co1—N4—C18	5.06 (15)	N3—C17—C18—N4	11.2 (3)
N1—Co1—N4—C18	−88.01 (15)	C16—C17—C18—N4	−172.5 (2)
C8—C7—N2—C11	0.2 (3)	C6—N4—C18—C17	−126.5 (2)
C8—C7—N2—Co1	179.25 (18)	C12—N4—C18—C17	107.4 (2)
O1—Co1—N2—C7	13.95 (19)	Co1—N4—C18—C17	−9.8 (2)
O2—Co1—N2—C7	102.28 (19)	N1—C5—C4—C3	−0.4 (4)
N3—Co1—N2—C7	−80.08 (19)	C6—C5—C4—C3	−177.5 (2)
N1—Co1—N2—C7	176.4 (4)	C2—C3—C4—C5	0.2 (4)
N4—Co1—N2—C7	−166.77 (19)	C44—C45—C46—C41	0.2 (4)
O1—Co1—N2—C11	−166.94 (15)	C42—C41—C46—C45	0.8 (4)
O2—Co1—N2—C11	−78.61 (15)	B1—C41—C46—C45	−176.6 (2)
N3—Co1—N2—C11	99.03 (16)	C12—N4—C6—C5	−154.53 (18)
N1—Co1—N2—C11	−4.5 (5)	C18—N4—C6—C5	79.3 (2)
N4—Co1—N2—C11	12.34 (15)	Co1—N4—C6—C5	−39.9 (2)
Co1—O2—C28—C27	−179.70 (16)	N1—C5—C6—N4	28.1 (3)
Co1—O2—C28—C19	0.2 (2)	C4—C5—C6—N4	−154.6 (2)
O1—C19—C28—O2	−9.4 (3)	C41—C42—C43—C44	1.5 (4)
C20—C19—C28—O2	172.02 (18)	C30—C29—C34—C33	−1.6 (4)
O1—C19—C28—C27	170.59 (18)	B1—C29—C34—C33	178.3 (2)
C20—C19—C28—C27	−8.0 (3)	C36—C35—C40—C39	0.3 (4)
C13—N3—C17—C16	−1.0 (3)	B1—C35—C40—C39	179.2 (2)
Co1—N3—C17—C16	176.53 (18)	C40—C35—C36—C37	−1.3 (4)
C13—N3—C17—C18	175.3 (2)	B1—C35—C36—C37	179.8 (2)
Co1—N3—C17—C18	−7.1 (3)	C47—C52—C51—C50	1.1 (4)
C15—C16—C17—N3	1.5 (4)	C11—C10—C9—C8	0.4 (4)
C15—C16—C17—C18	−174.5 (3)	C7—C8—C9—C10	0.5 (4)
C6—N4—C12—C11	149.20 (18)	C42—C43—C44—C45	−0.4 (4)
C18—N4—C12—C11	−84.9 (2)	C46—C45—C44—C43	−0.5 (4)
Co1—N4—C12—C11	34.6 (2)	C47—C48—C49—C50	−0.4 (4)
C24—C23—C22—C21	−0.8 (4)	C26—C25—C24—C23	1.0 (4)
O2—C28—C27—C26	−177.02 (19)	C22—C23—C24—C25	−1.6 (4)
C19—C28—C27—C26	3.0 (3)	C39—C38—C37—C36	0.7 (4)
C7—N2—C11—C10	0.7 (3)	C35—C36—C37—C38	0.8 (4)
Co1—N2—C11—C10	−178.43 (18)	N3—C13—C14—C15	2.2 (4)
C7—N2—C11—C12	−175.02 (19)	C48—C49—C50—C51	−1.0 (4)
Co1—N2—C11—C12	5.8 (2)	C52—C51—C50—C49	0.8 (4)
C9—C10—C11—N2	−1.0 (4)	C17—C16—C15—C14	−0.2 (4)
C9—C10—C11—C12	174.3 (2)	C13—C14—C15—C16	−1.6 (4)
N4—C12—C11—N2	−27.6 (3)	C31—C32—C33—C34	1.0 (4)
N4—C12—C11—C10	156.8 (2)	C29—C34—C33—C32	0.5 (4)
C28—C27—C26—C25	−173.8 (2)	C37—C38—C39—C40	−1.6 (4)
C28—C27—C26—C21	2.9 (3)	C35—C40—C39—C38	1.1 (4)
O1—C19—C20—C21	−171.60 (19)	C33—C32—C31—C30	−1.1 (4)
C28—C19—C20—C21	6.9 (3)	C34—C29—C30—C31	1.5 (4)
C21—C26—C25—C24	1.8 (3)	B1—C29—C30—C31	−178.5 (3)
C27—C26—C25—C24	178.5 (2)	C32—C31—C30—C29	−0.2 (4)
